# Supplementary material for: PICKLUSTER: a protein-interface clustering and analysis plug-in for UCSF ChimeraX
Source: Bioinformatics. 2023 Oct 16;39(11):btad629. doi: 10.1093/bioinformatics/btad629 (PMC10629935; doi:10.1093/bioinformatics/btad629)
Supplement: btad629_Supplementary_Data [file btad629_supplementary_data.pdf]

Supplementary Materials for

PICKLUSTER: A protein-interface clustering and analysis plug-in for UCSF ChimeraX

Luca R. Genz, Thomas Mulvaney, Sanjana Nair and Maya Topf

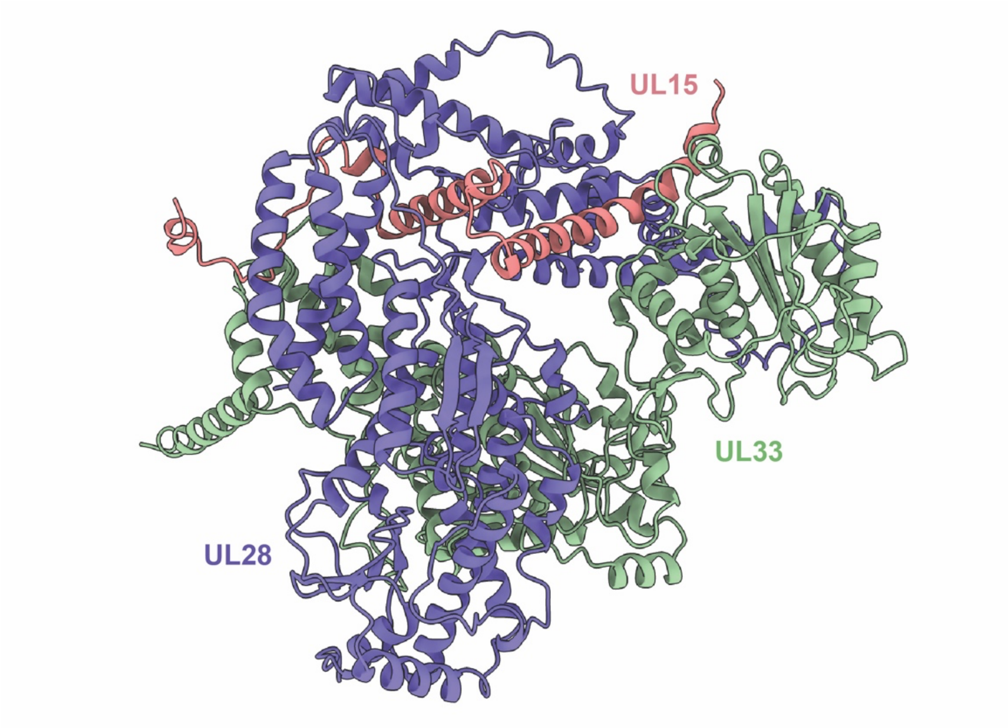

Fig. S1. Trimeric complex of UL28 (purple), UL15 (salmon) and UL33 (green) from *Epstein-Barr Virus* modelled with ColabFold (Mirdita *et al.*, 2022).
